# *Solanum nigrum* Unripe fruit fraction attenuates Adriamycin resistance by down-regulating multi-drug resistance protein (Mdr)-1 through Jak-STAT pathway

**DOI:** 10.1186/s12906-017-1872-3

**Published:** 2017-07-18

**Authors:** Sankar Jagadeeshan, Diana David, S. Jisha, S. Manjula, S. Asha Nair

**Affiliations:** 1 Cancer Research Program, Rajiv Gandhi Centre for Biotechnology, Thiruvananthapuram, Kerala, India; 2 Plant Molecular Biology, Rajiv Gandhi Centre for Biotechnology, Thiruvananthapuram, Kerala, India; 3 Department of Genetics, Dr. ALM Post Graduate Institute of Basic Medical Sciences, University of Madras, Taramani Campus, Chennai, Tamil Nadu India

**Keywords:** *Solanum nigrum*, Unripe fruits, Methanolic extract, Glycosidic fraction, Cancer, Adriamycin resistance

## Abstract

**Background:**

*Solanum nigrum*, herbal plant that commonly grows in temperate climate zone, has been used as a traditional folk medicine whose ripen fruits were proven to exhibit anti-tumor properties. In traditional Chinese medicine, it has been used for centuries to cure inflammation, edema, mastitis and hepatic cancer and in the Ayurvedic system of traditional medicine in India, *S. nigrum* is applied against enteric diseases, ulcer, diarrhea and skin diseases. A methanolic glycosidic extract fraction of unripe fruit of *S. nigrum* (SNME) was investigated for its anticancer property and possible mechanism to surmount adriamycin resistance in NCI/ADR-RES cells.

**Methods:**

The NCI/ADR-RES cells were treated with 7.8125, 15.625, 31.25, 62.5, 125 and 250 μg/ml of methanolic extract of *S. nigrum* (SNME) for 12, 24 and 48 h, to check the cell viability and proliferation. The cells were also exposed to adriamycin alone or in combination with SNME and the effects on cell growth were determined by MTT. Cell cycle analysis, Ethidium bromide and Acridine orange staining, Annexin-binding efficiency, nuclear condensation and DNA fragmentation of the apoptotic NCI/ADR-RES cells were also determined. To elucidate the relationship between SNME and multi drug resistance, we analyzed the expression levels of Mdr-1, JAK1, STAT3, and pSTAT3 in NCI/ADR-RES cells after treatment with SNME.

**Results:**

Results from the cytotoxicity assay showed a direct correlation between the concentration of methanolic glycosidic extract fraction of *S. nigrum* (SNME) and the surviving cell population. Combination with Adriamycin, SNME exhibits a synergistic action on NCI/ADR-RES cells, giving the first line of evidence to overcoming Adriamycin resistance. The SNME mediated cell growth suppression was proven to be apoptotic, based on results obtained from DNA fragmentation, annexin V apoptosis assaay and PARP cleavage analysis. Looking into the molecular insight SNME surpasses the chemoresistance of NCI/ADR-RES cells by inhibiting the JAK-STAT3 signaling pathway through the down regulation of JAK1, STAT3, pSTAT3, and Mdr1 expression.

**Conclusions:**

Collectively our findings suggest that unripe fruit of *Solanum nigrum* could possibly be used as a chemosensitizing agent against Adriamycin resistant cancers.

## Background

According to World Health Organization (WHO), death due to cancer is expected to increase 104% worldwide by the year 2020 with the largest increase (70%) predicted to occur in developing countries [1]. Chemotherapy is one of the most frequently used therapeutic modalities for cancer treatment. The development of resistance to chemotherapy poses a significant problem to patients who rely on conventional cytotoxic agents for the treatment of malignant disease. In recent years, there has been a global trend toward the isolation and identification of bioactive phytochemicals present in fruits, vegetables and herbs which possess substantial anti-carcinogenic properties [2]. Most of these bioactive substances exert their cancer chemotherapeutic activity by blocking cell cycle progression and triggering apoptotic cell death. Therefore, induction of apoptosis in tumor cells has become an indicator of the tumor treatment response in employing a plant derived bioactive substance to reduce and control human mortality due to cancer [3]. Recently, several studies have documented the ability of chemo-preventive phytochemicals to increase the sensitivity of cancer cells to anticancer drugs [4].

*Solanum nigrum,* an herbal plant that commonly grows in temperate climate zone, has been used as a traditional folk medicine because of its antiperiodic, antiphlogistic, diaphoretic, diuretic, emollient, febrifuge, narcotic, purgative and sedative effects. In traditional Chinese medicine, it has been used for centuries to cure inflammation, edema, mastitis and hepatic cancer [5]. In the ayurvedic system of traditional medicine in India, *S. nigrum* is applied against enteric diseases, ulcer, diarrhea and skin diseases.

Phytochemical investigations on *S. nigrum* whole plant reported that it contains alkaloids, flavonoids, tannins, saponins, glycosides, proteins, carbohydrates, coumarins and phytosterols and these bioactive principles shown to exhibit antitumor activity [6–9]. Son et al. [10], reported that ethanolic extract of *S. nigrum* has antiproliferative, apoptotic and cytotoxic effects on MCF- 7 cells. There are several reports which emphasize the role of extracts from *S. nigrum* berries as an antioxidant, antitumor, hepatoprotective, anti inflammatory and anti convulsant action [5, 11–15]. Even though vast literature study and experimental result analysis showed that *S. nigrum* employs various immunological applications in cancer, the beneficial role of its unripe berry extract on drug resistance in cancer has not yet been studied in detail.

Multi-drug resistance (Mdr1) gene, also known as ABCB1 gene, encodes an efflux transporter P-glycoprotein (P-gp) that limits a wide variety of drugs from penetrating cells and depositing them into the extracellular space [16, 17]. Since the MDR1 gene and P-gp were proved to induce drug resistance in certain tumors, pharmacogenetics concepts has had a significant impact on individual response of drug treatment and genotyping has been considered a new tool for predicting individual drug-metabolizing capabilities and therapeutic establishment [18]. Compelling evidence has now established that aberrant STAT3 expression has a critical role in the development, progression and drug resistance of human tumors. STAT3’s functions and its critical roles in tumorigenesis and tumor maintenance have qualified it as a valid target for the development of novel anticancer therapeutic modalities [19–21]. Several studies indicated a significant association between STAT3 expression and drug resistance in cancer. Inhibition of STAT3 activity enhanced chemosensitivity in hepatocellular carcinoma, stomach carcinoma and melanoma [22–24]. Additionally, some researchers demonstrated that multidrug resistance was consistent with STAT3 mRNA overexpression in cisplatin-resistant lung cancer cells [25] and STAT3 activity was found to be specifically elevated in drug-resistant neuroblastoma and ovarian cancer cells, while not in relevant drug sensitive cells [26]. Recently, it was demonstrated that inhibition of STAT3 effectively enhanced multidrug sensitivity via inhibiting Nanog/STAT3-mediated mdr1 gene expression in both MCF-7 cells and SK-OV-3.ipl cells [27]. Clinically, STAT3 is highly activated in drug non-sensitive advanced tumors. All these findings suggested that STAT3 might be associated with multi-drug resistance in various tumors and their relation need to be further explored.

Earlier studies done in our lab using methanolic extract of *S. nigrum* exhibited estrogen receptor (ER) dependent growth inhibition in MCF-7 cells and in vivo studies showed a classical uterotrophic response in ovariectomized mice elicited by *S. nigrum* [28]. Hence, this study was conducted to evaluate the chemo-sensitization efficacy of SNME on adriamycin resistant cancer cells (NCI/ADR-RES) and tried at deciphering the possible involvement of SNME on STAT3 mediated chemotherapy resistance in adriamycin resistant cancer cells (NCI/ADR-RES).

## Methods

### Plant material

Authentic certified seeds of *S. nigrum* (Acc No. IC 298650) procured from National Bureau of Plant Genetic Resources (NBPGR), Kerala Agricultural University Campus, Thrissur, Kerala, were planted and maintained in the green house of Rajiv Gandhi Centre for Biotechnology, Trivandrum, Kerala under uniform conditions of temperature and humidity.

### Phytochemical extraction

Mature, unripe fruits from 2 months old plants were collected, washed and oven dried at 60 °C. Uniformly dried fruits were powdered and 100 g of dried powder was used for soxhlet analysis. Extraction was carried out sequentially with 250 ml hexane and 250 ml chloroform at 50 °C for 18 h to remove the less polar lipid components and finally with 250 ml methanol at a temperature of 50 °C for 18 h to obtain the glycoside fraction. The defatted methanol fraction was further purified in silica gel glass column (Borosil) using the solvent mixture of 66 methanol: 33 chloroform: 1 glacial acetic acid as the mobile phase. The fractions were separately analyzed for the presence of glycosides by Thin Layer Chromatography (TLC) performed on pre-coated TLC plates (Silica gel 60 TLC Plates, Merck).

### HPLC analysis

The methanolic fraction was evaporated to dryness under vacuum using rota-evaporator (Buchi Lab Equipments, USA) and stored at 4 °C for HPLC analysis. For preparation of standard, 10 mg of α-solanine (Sigma, USA) was weighed and added to a 10 mL volumetric flask, and made up to 10 mL with methanol (HPLC grade, Merck). 100 mg of the extract was weighed and made up to 100 mL, followed by filtration through a Millex syringe-driven filter unit (Millipore Corporation, Bedford, USA) before injection. The sample injection volume was 2.0 μl and the chromatogram was recorded at 210 nm. HPLC was performed in model LC-10AT vp (Shimadzu Corporation, Kyoto, Japan) using C18 column (3 μm size, 5 cm × 2.1 mm in length; Supelco, USA) with water-acetonitrile (HPLC grade; Merck, Darmstadt, Germany) as the mobile phase. Separation was carried out at a flow rate of 0.2 mL/min.

### Cell culture

NCI/ADR-RES cell line was obtained from National Cancer Institute (NCI, USA). The cells were maintained in DMEM (Sigma, USA) containing 10% heat inactivated FBS (GIBCO, USA) and 1% antibiotic-antimycotic cocktail (GIBCO, USA).

### Cell viability assay

Briefly, cells (5000 cells/well) were seeded into a 96 well plate and incubated for 24 h. After 24 h, cells were replenished with fresh medium containing different concentration of compound. After 48 h of incubation, MTT (500 μg/ml final concentration, Sigma, USA) was added to each well and incubated for 4 h in order to allow the conversion of MTT to formazan crystals. Finally, the formazan crystals formed were dissolved in isopropyl alcohol and the OD was measured at 570 nm using ELISA microplate reader (Bio Rad, USA). The amount of formazan crystals formed is directly proportional to the viability of cells and the percentage growth inhibition by the compound was calculated and tabulated.

### Combination treatment

NCI/ADR RES cells were exposed to adriamycin alone or in combination with SNME, for 48 h, and the effects on cell growth were determined by MTT. Synergistic efficacy was determined by the isobologram and combination-index methods of Chou and Talalay (CompuSyn Software [29, 30],).

### Cell cycle analysis

Cell cycle progression was identified by measuring the DNA content. Briefly, cells after treatment, were trypsinized, washed twice in PBS and fixed using 70% of ice cold ethanol for 30 min. Fixed cells were washed twice and treated with 200 μg/ml of RNase A at 37 °C for 1 h. Finally propidium iodide was used to stain the cells. Analysis was done in FACS Aria (BD, Mountain View, CA, USA) using BD FACS Diva software.

### Apoptotic assays

2 × 10^5^ cells were seeded in a 35 mm culture dish and SNME treatment was given at 125 μg/ml for 24 H*. medium* was removed and the cells were washed with phosphate buffered saline. The cells were then given a combined staining of acridine orange (50 mg/ml) and ethidium bromide (5 mg/ml) for 5 min at room temperature, and examined by an inverted fluorescence microscope. Similar method was followed for Hoechst staining (5 mg/ml) except for the final step in which the cells were given an incubation period of 15 min at 37 °C before visualization.

For annexin-binding assay, 1 × 10^6^ cells were seeded in 60 mm culture dishes. SNME treatment was given (125 μg/ml for 12 h and 24 h) and the cells were harvested and stained with FITC-labeled annexin using Annexin V-FITC Apoptosis Detection Kit according to the manufacturer’s instruction, and a flow cytometric analysis was carried out using FACS Aria (Special order system, BD, USA).

### DNA fragmentation assay

1 × 10^6^ cells were harvested after SNME treatment for 24 h, washed, and incubated in 20 μl of 50 mM Tris-HCl (pH 8.0), 10 mM EDTA, 0.5% SDS, and 0.5 μg/ml proteinase K (Sigma) for 1 h at 50 °C. 10 μl of 0.5 μg/ml RNase A was added and incubated for 1 h. The digested samples were incubated with 10 μl of 10 mM EDTA (pH 8.0), 0.25% bromophenol blue, and 40% sucrose at 70 °C. The DNA was separated in 2% agarose gel and visualized by ultraviolet (UV) illumination after ethidium bromide staining.

### Immunoblot analysis

2 × 10^6^ cells were seeded in 100-mm culture dishes and SNME was given at different concentrations (7.8, 15.6, 31.2, 62.5, and 125 μg/ml) and time periods (6, 12, 24 and 48 h). Cells were then lysed using lysis buffer comprising 10% NP40, 5 M NaCl, 1 M HEPES, 0.1 M DTT, 0.1 M EGTA, 0.1 M EDTA, protease inhibitors (Sigma, USA) and total cell extract was obtained by differential centrifugation (14,000 rpm for 10 min). The protein concentrations were determined using Bradford’s assay and 60 μg of proteins were resolved by 10% SDS-PAGE, and the separated proteins were electrotransferred onto nitrocellulose membrane (Amersham Pharmacia Biotech, USA). After pre-blocking these membranes with 5% skimmed milk, they were treated with antibodies against STAT3 (1:200, Santa Cruz Biotechnology, USA), pSTAT3 (Tyr 705) (1:200, Santa Cruz Biotechnology, USA), pSTAT3 (Ser 727) (1:100, Cell Signaling, USA), JAK1 (1:300, Cell Signaling, USA), PARP (1:100, Cell Signaling, USA), Mdr1 (1:100, Santa Cruz Biotechnology, USA), and β- actin (1:5000, Sigma, USA) as primary antibodies and incubated overnight at 4 °C. Horseradish peroxidase-conjugated anti-rabbit (1:5000, Santa Cruz Biotechnology, USA) and anti-mouse (1:5000, Santa Cruz Biotechnology, USA) antibodies were used as secondary antibodies and incubated for 1 h at room temperature. Immunoreactive bands were developed with an ECL system (Amersham Pharmacia Biotech, Uppsala, Sweden).

## Result

### HPLC analysis

**Fig. 1:**
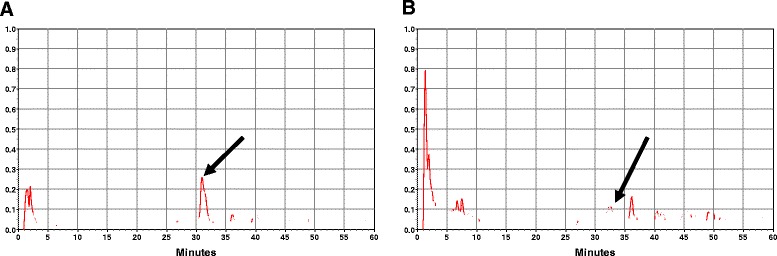
**a**, HPLC chromtogram of α-solanine. **b**, HPLC chromatogram of SNME fraction. Arrow indicates the peak of α-solanine

The retention time for α-solanine was around 30 min (Fig. 1a). A comparable peak was observed for the extract, which indicates the presence of α -solanine in the glycoside fraction (Fig. 1b). The presence of α-solanine which is the main glycoside, was also confirmed by TLC. The additional peaks indicate the presence of more glycosides. FAB/MS (Jeol SX-102, Japan) was performed with the glycoside fractions, which indicated the presence of two major peaks representing glycosides with molecular weights 884.06 and 884, which coincides with those of known glycosides like solasonine and solamargine respectively. Altogether, our results suggest the presence of at least three known *Solanum* glycosides viz. α-solanine, solasonine and solamargine in the extract. However, detailed analysis could not be performed due to lack of availability of these solasonine and solamargine standards.

### SNME inhibits cell proliferation

**Fig. 2:**
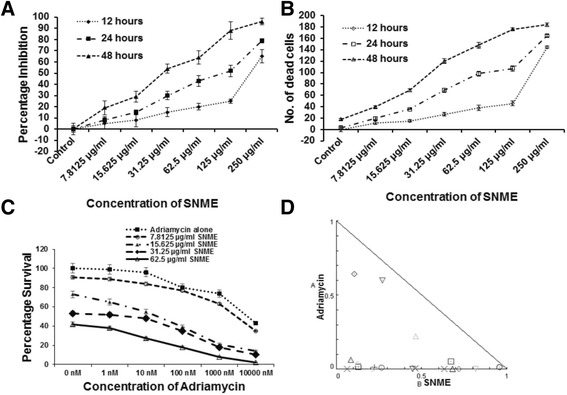
**a**, Effects of SNME on NCI/ADR-RES cell proliferation. Cells were treated with 7.8125, 15.625, 31.25, 62.5, 125 and 250 μg/ml of methanolic extract for 12, 24 and 48 h. Control cells were maintained in the vehicle for the indicated time periods. The percentage inhibition increases as the concentration of SNME increases and the IC_50_ value obtained was 125 μg/ml at 24 h. Results are represented as mean ± SEM of three assays. **b**, Effects of SNME on NCI/ADR-RES cell viability. Cells were treated with 7.8125, 15.625, 31.25, 62.5, 125 and 250 μg/ml of methanolic extract for 12, 24 and 48 h. Control cells were maintained in the vehicle for the indicated time periods. Results are represented as mean ± SEM of three assays. **c**, Effects of Adriamycin and Adriamycin–SNME combination treatment on NCI/ADR-RES cell proliferation. Cells were treated with increasing concentration of Adriamycin (1 nM-10,000 nM) with SNME ranging from 7.8125-62.5 μg/ml of methanolic extract for 48 h. Control cells were maintained in the vehicle for the indicated time periods. There was a synergistic action making the NCI/ADR RES cell lines sensitive to Adriamycin treatment in combination with SNME. Results are represented as mean ± SEM of three assays. **d**, Isobologram analysis of NCI/ADR RES cell lines in combination treatment with Adriamycin and SNME. Data calculated from surviving fractions following 48 h drug incubation. The diagonal line represents the isoeffect line of additivity. Each point is the mean determined from experiments performed in triplicate. Combination index (CI) values calculated using Compusyn software is represented by points above (indicate antagonism between drugs) or below the lines (indicate synergy)

We first examined the inhibitory action of SNME on NCI/ADR-RES cells by MTT assay. NCI/ADR-RES cells were treated with serial concentrations of SNME for different time intervals as shown in Fig. 2a. SNME inhibited cell proliferation in a dose and time dependent manner. We could attain a 50% inhibition at about 125 μg/ml of the SNME for about 24 h. On prolonged incubation of SNME for 48 h, the percentage inhibition rose with increase in concentration. Similar pattern of result was obtained on cell viability analysis using trypan blue assay (Fig. 2b).

### SNME synergistically act with Adriamycin on combination treatment

To determine whether the SNME sensitizes Adriamycin resistant NCI/ADR RES cells to Adriamycin, the cells were treated with varying concentration of Adriamycin in combination with varying concentration of SNME (Fig. 2c). Synergy was evaluated using the CompuSyn Software and observed at multiple drug concentrations resulting in combination indices under 0.5 at *Fa* of 0.5 (50% reduction of cell growth) (Fig. 2d). Combination treatment of SNME with Adriamycin indicate a synergistic action which sensitizes the cells and enhances the efficacy of the Adriamycin to kill the cancerous drug resistant cells.

### SNME promotes sub G1 phase accumulation

**Fig. 3:**
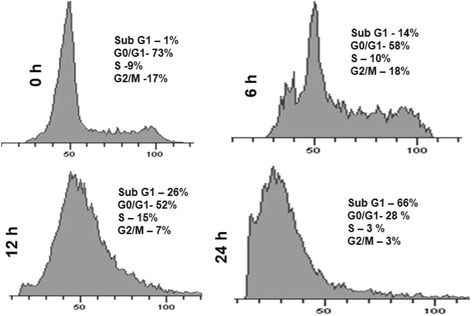
Effects of SNME on cell division cycle of asynchronously growing NCI/ADR-RES cells. Cells were treated with 125 μg/ml of methanolic extract for 0, 6, 12 and 24 h, stained with propidium iodide and analyzed by flow cytometry for DNA content. An increase in Sub G_0_ phase indicated DNA fragmentation, a prerequisite for apoptosis

To determine whether the SNME mediated inhibition of cell proliferation was the result of cell cycle arrest induced apoptosis; we analyzed the DNA content of cells by propidium iodide staining followed by flow cytometry analysis. The NCI/ADR-RES cells were treated with 125 μg/ml of SNME for different time intervals ranging from 6 h to 24 h resulting in the accumulation of cells at sub G1 phase clearly indicating the onset of cell death (Fig. 3).

### SNME triggers apoptosis

**Fig. 4:**
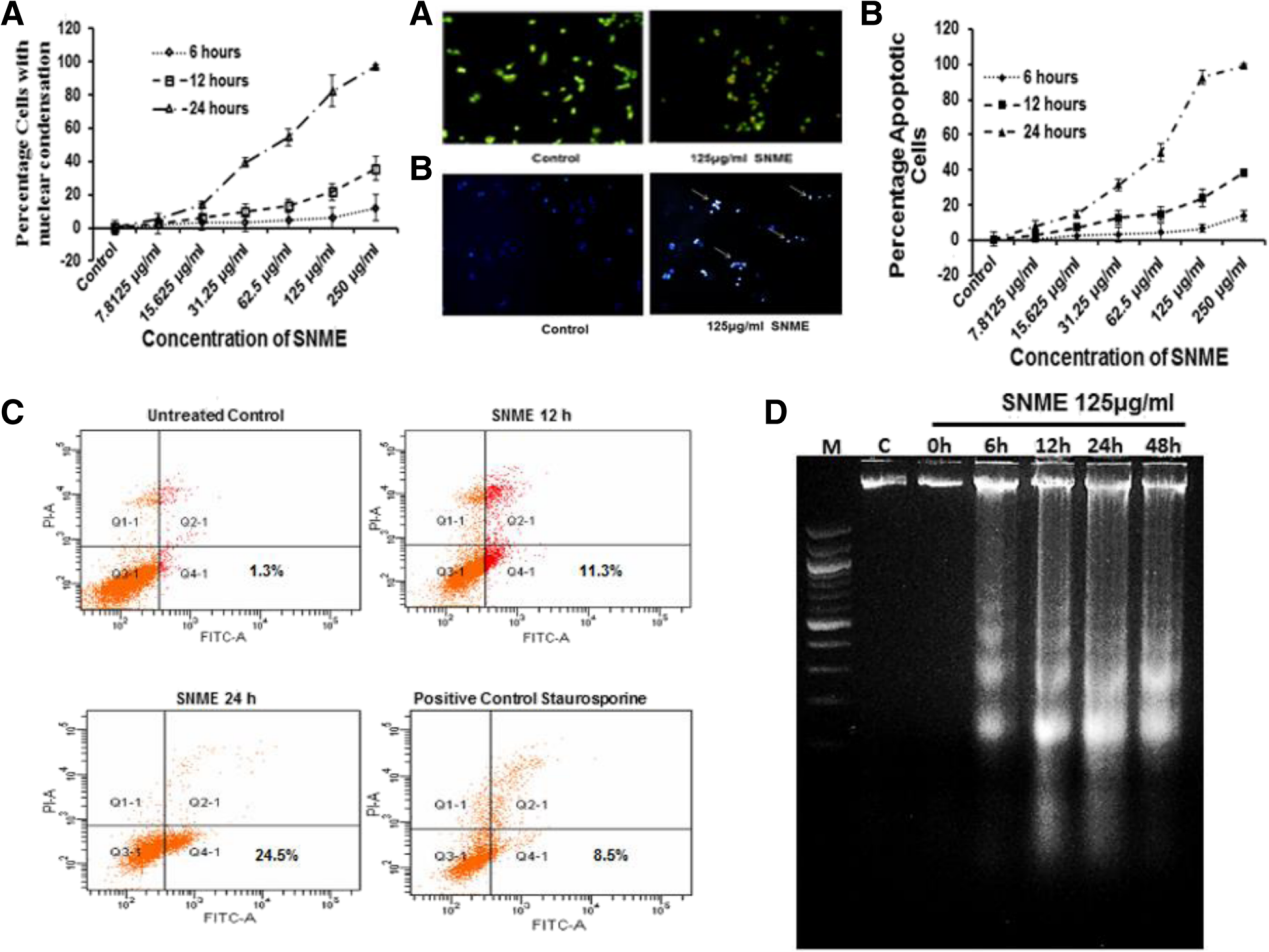
**a**, Apoptotic potential of SNME on NCI/ADR-RES cells. Cells were treated with 7.8125, 15.625, 31.25, 62.5, 125 and 250 μg/ml of methanolic extract for 6, 12 and 24 h, stained with ethidium bromide-acridine orange. Control cells were maintained in the vehicle for the indicated time periods. Results are represented as mean ± SEM of three assays. Representative microscopic image showing ethidium bromide – acridine orange stained apoptotic cells after treatment with 125 μg/ml SNME on NCI/ADR-RES cells. **b**, Nuclear condensation studies of SNME on NCI/ADR-RES cells. Cells were treated with 7.8125, 15.625, 31.25, 62.5, 125 and 250 μg/ml of methanolic extract for 6, 12 and 24 h, stained with Hoescht. The number of bright blue condensed spots indicating apoptotic cells with condensed chromatin increases with *Solanum nigrum* treatment in a time and concentration dependent manner. Control cells were maintained in the vehicle for the indicated time periods. Results are represented as mean ± SEM of three assays. Representative microscopic image showing nuclear condensation (→) in Hoescht stained apoptotic cells after treatment with 125 μg/ml SNME on NCI/ADR-RES cells. **c**, SNME induces apoptosis in NCI/ADR-RES cell. FITC-conjugated annexin binding to phosphatidyl serine, exposed to the outer leaflet, is measured by FACS analysis in NCI/ADR-RES cells after treatment with methanolic extract of *S. nigrum* 125 μg/ml for 12 h and 24 h. Apoptotic population (Q4) FACS histogram plotted against PI vs. FITC staining with a notable increase in the number of apoptotic cells after treatment for 24 h. Staurosporine (1 μg/ml) treated cells were used as positive control. **d**, DNA fragmentation of NCI/ADR-RES cells exposed to methanolic extract of unripe *Solanum nigrum*. Cells were incubated with 125 μg/ml at 0, 6, 12, 24 and 48 h. DNA ladders reflecting the presence of DNA fragments were viewed on ethidium –bromide stained 2% agarose gel

Morphological changes associated with apoptosis were evaluated by using phase contrast microscopy. Hoescht staining and ethidium bromide-acridine orange dual staining (Fig. 4a) was used to visualize nuclear condensation and onset of apoptosis. Vehicle treated cells were intact and homogeneously stained. Cells treated with SNME showed nuclear condensation. The number of cells with condensed nucleus increased with dose and time as evident in Fig. 4b.

**Fig. 5:**
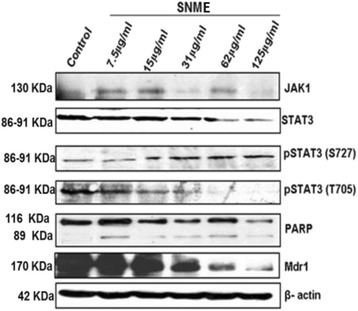
Western blot analysis of Jak1, Stat3, pStat3 (S727), pStat3 (T705), PARP and Mdr1 protein levels in NCI/ADR-RES cells treated with 7.5, 15, 31, 62 and 125 μg/ml of SNME for 24 h. β-actin was used to verify equal gel loading

Further to confirm SNME induce apoptosis, we performed Annexin V FITC staining. There was a notable increase (24.5%) in the number of apoptotic population after treatment with SNME -125 μg/ml for 24 h (Fig. 4c). The DNA fragmentation assay also showed laddering of DNA at various concentrations (Fig. 4d) of SNME giving evidence to its apoptotic potential. Cleavage of PARP was also observed on dose and time dependent treatment of SNME on NCI/ADR-RES cells (Fig. 5a).

### SNME down regulates the expression of Mdr-1

To elucidate the relationship between STAT3 and multi drug resistance, we observed the expression pattern of Mdr-1, the multidrug resistance protein that help in drug efflux mechanism, after treatment with SNME*.* As shown in Fig. 5a, the expression of Mdr-1 was found to be decreased in a dose dependent manner after treatment with SNME. This probably indicates that cytotoxicity of NCI/ADR-RES cells was elevated owing to STAT3 inhibition.

### SNME treatment down regulates STAT3 expression

The activity of STAT3 is usually higher in MDR tumors, and inhibition of STAT3 activity might reverse chemoresistance (Duan et al. [26]). In this study, we looked for the effect of SNME on STAT3 expression. It was observed that treatment with SNME decreased the expression of STAT3 in a dose dependent manner, along with JAK1. The expression of phosphorylated STAT3 (Tyr705) was also found to be decreased in a concomitant manner (Fig. 5). Interestingly there was not much variation in the expression level of phosphorylated STAT3 (ser727) after treatment with SNME (Fig. 5). These results suggest that deactivation of STAT3 could increase drug accumulation by suppressing Mdr1 expression.

## Discussion

The effectiveness of chemotherapy is seriously limited by multi-drug resistance which is mediated mainly by P-gp and Mdr-1. Since the early 1980s, some compounds were found to overcome P-gp-mediated MDR. However, they had only limited success in clinical trials. Therefore, the characterization of signaling pathways sustaining MDR is thus essential for designing rational novel therapies [31]. This notion is supported by the data in the present study showing that inhibition of STAT3 pathway down regulated Mdr-1 expression in adriamycin resistant NCI/ADR-RES cells.

The main goal of this study was to investigate the effect of SNME of unripe *S. nigrum* fruits on cell growth and apoptosis induction in adriamycin resistant cell line NCI/ADR-RES. *S. nigrum* being a minor food crop of *Solanaceae* family, our preliminary results attributing its antineoplastic function warrants a deeper molecular understanding on the implication of *solanum* glycosides as a whole in chemo-sensitization. Initially, we observed that treatment with *S. nigrum* dramatically induced active inhibition of DNA synthesis. This result is in agreement with a previous study showing that *S. nigrum* exhibited anti-neoplastic effect on several human tumor cell lines [32]. In addition, since number of cells stained with MTT or trypan blue excluded were decreased with increased time and dose of the treatment (Fig. 2a and b), we hypothesized that SNME mediated inhibition of DNA synthesis may be exerted through a cytotoxic effect, rather than a cytostatic effect. Subsequently, we analyzed whether the cytotoxic effect is mediated via an apoptotic pathway. As evidenced by the presence of increased number of positively stained cells in Annexin V assay (Fig. 4c), PARP cleavage (Fig. 5) and the characteristic fragmentation of nuclear DNA (Fig. 4d) after SNME treatment, it appeared that apoptosis was the main mechanism for the cytotoxic effect of SNME on NCI/ADR-RES cells. Since it has been suggested that apoptosis plays a critical role in tissue homeostasis and cancer development, the modulation of apoptosis has become an interesting target for both therapeutic and preventive approaches to cancer treatment [33].

Accumulating evidence supported that activated STAT3 might be a target for anti-tumor treatment. In this study, we observed that expression of STAT3 and JAK1 was down regulated in SNME treated cells in a time and concentration dependent manner (Fig. 5a). JAK-STAT signaling is an important transduction pathway between cell survival and apoptosis. JAK1-dependent STAT3 activation has been reported to promote tumor cell cycling, survival, and invasiveness, enhance telomerase activity and modulate angiogenesis [19, 21, 34, 35]. It is generally accepted that the tyrosine phosphorylation of STATs is a prerequisite for their DNA binding and transactivation, although growth factors and cytokines induce phosphorylation of STATs on both tyrosine and serine. Earlier studies have shown that serine phosphorylation is required for the DNA binding of Stat3 in certain cell types. However, it was demonstrated later that phosphorylation on Ser-727 is not necessary for its DNA binding, but is required for the full transcriptional activity of Stat1 and Stat3. On the other hand, a negative effect of Ser-727 phosphorylation on the tyrosine phosphorylation of Stat3 has also been suggested [36]. In addition it is reported that phospho-S727 has an intrinsic mechanism for shortening the duration of STAT3 activity specifically by enhancing dephosphorylation of phospho-Tyr705 [37, 38]. The STAT3 signaling cascade is frequently activated in cancer cells and results in enhanced resistance of these cells to apoptosis through multiple mechanisms [39]. Given the substantial biological and molecular evidence supporting STAT3 as a valid target and the increasing number of human tumors that harbor constitutively-active STAT3, novel anticancer therapeutic modalities based on STAT3 inhibition will have widespread therapeutic applications. Thus *Solanum nigrum* can either be used as standalone agent or in combination with chemotherapy or other molecular-targeted therapeutic agents. Down regulation of these genes by *Solanum nigrum* also could contribute to this compound’s potent cell cycle arrest and apoptotic effect on cancer cells.

Furthermore, Zhang et al. [31], reported that dephosphorylation of STAT3 reverses chemotherapeutics resistance of leukemia cells via down-regulating P-gp. In our study, we observed that treatment of NCI/ADR-RES cells with SNME down regulated the expression of Mdr-1 (Fig. 5a) which may also contribute to its cytotoxic effect. Several studies aimed to modulate MDR1 gene and P-gp expression to improve the effects of some drugs, increasing the efficacy of treatments of certain diseases [40]. Modulation of P-gp can affect drug bioavailability, increase or decrease penetration of its substrates into the central nervous system, and affect the therapeutic efficacy [17, 41, 42]. Bourguignon et al. [27], reported that Nanog complexes with STAT3 can transactivate Mdr1 gene. However, in our study we observed inhibition of STAT3 expression, which leads to delayed STAT3 mediated transactivation of Mdr1 gene contributing to its down regulation. In summary, the present study elucidated a novel role of unripe *S. nigrum* fruit extract in inhibiting cellular proliferation and promoting apoptosis in Adriamycin resistant cell line NCI/ADR-RES, which further suggested that STAT3 could be a potential target in modulating drug resistant cancers.

## Conclusion

The effectiveness of chemotherapy is seriously limited by multi-drug resistance, mediated mainly by P-glycoprotein (P-gp) and multi-drug resistance protein 1 (Mdr-1). In our study, we analyzed the effect of methanolic extract of unripe fruits of *S. nigrum* in combating multi-drug resistance (Mdr-1) expression and inducing apoptosis in Adriamycin resistant cells (NCI/ADR-RES) via the JAK-STAT pathway. The higher activity of STAT3 is usually associated within multi-drug resistant (MDR) tumors, and inhibition of STAT3 activity might reverse chemo-resistance. Our findings suggest that methanolic extract of unripe fruits of *S. nigrum* could be used as a chemo-sensitizing agent for Adriamycin resistant cancer cells and there by surpass chemo-resistance via inhibiting the JAK-STAT3 pathway.

## Abbreviations

ABCB1: ATP Binding Cassette Subfamily B Member 1
ADR-RES: Adriamycin Resistance
FITC: Fluorescein isothiocyanate
HPLC: High Performance Liquid Chromatography
JAK1: Janus Kinase 1
Mdr: Multi drug resistance
NCI: National Cancer Institute
OD: Optical Density
PARP: Poly ADP Ribose Polymerase
P-gp: P glycoprotein
SNME: *Solanum nigrum* Methanolic Extract
STAT: Signal Transducer and Activator of Transcription

## References

[1] Ferlay J, Soerjomataram I, Ervik M, Dikshit R, Eser S, Mathers C, Rebelo M, Parkin DM, Forman D, Bray F (2013). GLOBOCAN 2012. Cancer incidence and mortality worldwide: IARC Cancer Base no. 11.

[2] Ashraf RA, Sarfraz A, Mahmood A, Din MU (2015). Chemical composition and in vitro antioxidant and antitumor activities of *Eucalyptus camaldulensis Dehn.* Leaves. Ind Crop Prod.

[3] Paschka AG, Butler R, Young CYF (1998). Induction of apoptosis in prostate cancer cell lines by the green tea component, β-epigallocatechin-3-gallate. Cancer Lett.

[4] Nabekura T, Kamiyama S, Kitagawa S (2005). Effects of dietary chemopreventive phytochemicals on P-glycoprotein function. Biochem Biophys Res Commun.

[5] Kumar VP, Shashidhara S, Kumar MM, Sridhara BY (2001). Cytoprotective role of *Solanum nigrum* against gentamicin-induced kidney cell (Vero cells) damage in vitro. Fitoterapia.

[6] Heo KS, Lee SJ, Ko JH, Lim K, Lim KT (2004). Glycoprotein isolated from *Solanum nigrum* inhibits the DNA-binding activities of NF-κB and AP-1, and increases the production of nitric oxide in TPA stimulated MCF-7 cells. Toxicol in Vitro.

[7] An L, Tang J, Liu XM, Gao NN (2006). Review about mechanisms of anti-cancer of *Solanum nigrum*. Zhongguo Zhong Yao Za Zhi.

[8] Zhou X, He X, Wang G, Gao H, Zhou G, Ye W, Yao X (2006). Steroidal saponins from *Solanum nigrum*. J Nat Prod.

[9] Ji YB, Gao SY, Ji CF, Zou X (2008). Induction of apoptosis in HepG2 cells by solanine and Bcl-2 protein. J Ethnopharmacol.

[10] Son YO, Kim J, Lim JC, Chung Y, Chung GH, Lee JC (2003). Ripe fruits of *Solanum nigrum L*. inhibit cell growth and induce apoptosis in MCF-7 cells. Food Chemical Toxicology.

[11] Lee JS, Lim KK, Lim KT (2005). 150 kDa glycoprotein isolated from *Solanum nigrum Linne* enhances activities of detoxicant enzymes and lowers plasmic cholesterol in mouse. Pharmacol Res.

[12] Lim KT (2005). Glycoprotein isolated from *Solanum nigrum L* kills HT-29 cells through apoptosis. J Med Food.

[13] Li J, Li QW, Feng T, Zhang T, Li K, Zhao R, Han Z, Gao D (2007). Antitumor activity of crude polysaccharides isolated from *Solanum nigrum Linne* on U14 cervical carcinoma bearing mice. Phytother Res.

[14] Lin HM, Tseng HC, Wang CJ, Chyau CC, Liao KK, Peng PL, Chou FP (2007). Induction of autophagy and apoptosis by the extract of *Solanum nigrum* Linn in HepG2 cells. J Agric Food Chem.

[15] Ravi V, Saleem TSM, Patel SS, Ramamurthy J, Gauthaman K (2009). Anti-inflammatory effect of Methanolic extract of *Solanum nigrum Linn* berries. Int J Appl Res Nat Prod.

[16] Xie R, Hammarlund-Udenaes M, de Boer AG, de Lange EC (1999). The role of P- glycoprotein in blood-brain barrier transport of morphine: transcortical microdialysis studies in MDR1a (−/−) and MDR1b (+/+) mice. Br J Pharmacol.

[17] Wang JS, Ruan Y, Taylor RM, Donovan JL, Markowitz JS, DeVane CL (2004). Brain penetration of methadone (R)- and(S)- enantiomers is greatly increased by P- glycoprotein deficiency in the blood-brain barrier of Abcb1a gene knockout mice. Psychopharmacology.

[18] Ieiri I, Takane H, Otsubo K (2004). The MDR1 (ABCB1) gene polymorphism and its clinical implications. Clin Pharmacokinet.

[19] Buettner R, Mora LB, Jove R (2002). Activated STAT signaling in human tumors provides novel molecular targets for therapeutic intervention. Clin Cancer Res.

[20] Turkson J, Jove R (2000). STAT proteins: novel molecular targets for cancer drug discovery. Oncogene.

[21] Yu H, Jove R (2004). The STATS of cancer-new molecular targets come of age. Nat Rev Cancer.

[22] Lau CK, Yang ZF, Lam SP, Lam CT, Ngai P, Tam KH, Poon RT, Fan ST (2007). Inhibition of Stat3 activity by YC-1 enhances chemo-sensitivity in hepatocellular carcinoma. Cancer Biol Ther.

[23] Sredni B, Weil M, Khomenok G, Lebenthal I, Teitz S, Mardor Y, Ram Z, Orenstein A, Kershenovich A, Michowiz S, Cohen YI, Rappaport ZH, Freidkin I, Albeck M, Longo DL, Kalechman Y (2004). Ammonium trichloro (dioxoethylene-o,o’) tellurate (AS101) sensitizes tumors to chemotherapy by inhibiting the tumor interleukin 10 autocrine loop. Cancer Res.

[24] Zhou J, Ong CN, Hur GM, Shen HM (2010). Inhibition of the JAK-STAT3 pathway by andrographolide enhances chemosensitivity of cancer cells to doxorubicin. Biochem Pharmacol.

[25] Ikuta K, Takemura K, Kihara M, Nishimura M, Ueda N, Naito S, Lee E, Shimizu E, Yamauchi A (2005). Overexpression of constitutive signal transducer and activator of transcription 3 mRNA in cisplatin-resistant human non-small cell lung cancer cells. Oncol Rep.

[26] Duan Z, Foster R, Bell DA, Mahoney J, Wolak K, Vaidya A, Hample C, Lee H, Seiden MV (2006). Signal transducers and activators of transcription 3 pathway activation in drug-resistant ovarian cancer. Clin Cancer Res.

[27] Bourguignon LY, Peyrollier K, Xia W, Gilad E (2008). Hyaluronan-CD44 interaction activates stem cell marker Nanog, Stat-3-mediated MDR1 gene expression, and ankyrin-regulated multidrug efflux in breast and ovarian tumor cells. J Biol Chem.

[28] Jisha S, Sreeja S, Manjula S (2011). In vitro and in vivo estrogenic activity of glycoside fractions of *Solanum nigrum* fruits. Indian J Med Res.

[29] Chou TC (2010). Drug combination studies and their synergy quantification using the Chou-Talalay method. Cancer Res.

[30] Chou TC, Martin N (2005). CompuSyn for drug combinations: PC software and User’s guide: a computer program for Quantitation of synergism and antagonism in drug combinations, and the determination of IC50 and ED50 and LD50 values.

[31] Zhang X, Xiao W, Wang L, Tian Z, Zhang J (2011). Deactivation of signal transducer and activator of transcription 3 reverses chemotherapeutics resistance of leukemia cells via down-regulating P-gp. PLoS One.

[32] Hu K, Kobayashi H, Dong A, Jing Y, Iwasaki S, Yao X (1999). Antineoplastic agents III: steroidal glycosides from *Solanum nigrum*. Planta Med.

[33] Ahmad N, Feyes DK, Nieminen AL, Agarwal R, Mukhtar H (1997). Green tea constituent epigallocatechin-3-gallate and induction of apoptosis and cell cycle arrest in human carcinoma cells. J Nat Cancer Institute.

[34] Bowman T, Garcia R, Turkson J, Jove R (2000). STATs in oncogenesis. Oncogene.

[35] Turkson J (2004). STAT proteins as novel targets for cancer drug discovery. Expert Opin Ther Targets.

[36] Lim PC, Cao X (1999). Serine phosphorylation and negative regulation of Stat3 by JNK. J Biol Chem.

[37] Chung J, Uchida E, Grammer CT, Blenis J (1997). STAT3 serine phosphorylation by ERK-dependent and independent pathways negatively modulates its tyrosine phosphorylation. Mol Cell Biol.

[38] Wakahara R, Kunimoto H, Tanino K, Kojima H, Inoue A, Shintaku H, Nakajima K (2012). Phospho-Ser727 of STAT3 regulates STAT3 activity by enhancing dephosphorylation of phospho-Tyr705 largely through TC45. Genes Cells.

[39] Siddiquee AL, Zaid K, Turkson J (2008). STAT3 as a target for inducing apoptosis in solid and hematological tumors. Cell Res.

[40] Mickisch GH, Pastan I, Gottesman MM (1991). Multidrug resistant transgenic mice as a novel pharmacologic tool. BioEssays.

[41] Mizuno N, Niwa T, Yotsumoto Y, Sugiyama Y (2003). Impact of drug transporter studies on drug discovery and development. Pharmacol Rev.

[42] Dagenais C, Graff CL, Pollack GM (2004). Variable modulation of opioid brain uptake by P-glycoprotein in mice. Biochem Pharmacol.

